# Acceptability, usability, and willingness to pay for HIV self‐test kits distributed through community‐based, PLHIV network‐led and private practitioners models in India: Results from the STAR III Initiative

**DOI:** 10.1002/jia2.26348

**Published:** 2024-08-08

**Authors:** Chinmay Laxmeshwar, Asha Hegde, Alpana Dange, Kannan Mariyappan, Manish Soosai, Sandeep Mane, Murugesan Sivasubramanian, Mahesh Doddamane, Madhuri Mukherjee, G. S. Shreenivas, Manoj Pardesi, Vinod Jambhale, Venkateswara Rao Pakkela, Vijayaraman Arumugam, Vedant Rungta, Yashika Bansal, Jatin Chaudary, Vijay Yeldandi, Mahalingam Periasamy, Chengappa Uthappa, Sudhir Chawla, Sunita Upadhyaya, Melissa Nyendak, Venkatesan Chakrapani, Sheela Godbole, Vinita Verma, Bhawani Singh Kushwaha, Chinmoyee Das, Shobini Rajan, Anoop Kumar Puri, J. V. R. Prasada Rao, Tarun Bhatnagar, D. C. S. Reddy, Kimberly Green

**Affiliations:** ^1^ PATH Mumbai India; ^2^ The Humsafar Trust Mumbai India; ^3^ Solidarity and Action Against The HIV Infection in India (SAATHII) Chennai India; ^4^ International Training and Education Centre for Health (I‐TECH) New Delhi India; ^5^ National Coalition of People Living with HIV in India (NCPI+) New Delhi India; ^6^ Network of Maharashtra By People Living With HIV/AIDS (NMP+) Pune India; ^7^ Voluntary Health Services (VHS) Chennai India; ^8^ William J. Clinton Foundation New Delhi India; ^9^ Society for Health Allied Research & Education India (SHARE India) Hyderabad India; ^10^ Division of Global HIV and TB Centres for Disease Control and Prevention (CDC) New Delhi India; ^11^ Centre for Sexuality and Health Research and Policy (C‐SHaRP) Chennai India; ^12^ Indian Council of Medical Research National AIDS Research Institute (ICMR‐NARI) Pune India; ^13^ National AIDS Control Organisation Ministry of Health and Family Welfare, Government of India New Delhi India; ^14^ Former Health Secretary Government of India New Delhi India; ^15^ Indian Council of Medical Research National Institute of Epidemiology (ICMR‐NIE) Chennai India; ^16^ Independent Consultant Lucknow India; ^17^ PATH Hanoi Vietnam

**Keywords:** HIV/AIDS, HIV testing, HIV self‐testing, operational research, key population, India

## Abstract

**Introduction:**

HIV self‐testing (HIVST) has been shown to increase the uptake of HIV testing and help achieve the UNAIDS 95‐95‐95 targets. This study assessed the acceptability, usability (ease of use and result interpretation) and the willingness to pay for HIVST kits distributed through three distribution models, namely the community‐based, PLHIV network‐led and private practitioners models, in India.

**Methods:**

This cross‐sectional study was implemented across 14 states in India between September 2021 and June 2022. All participants could choose between blood‐based or oral‐fluid‐based test kits. Participants were shown a test‐kit usage demonstration video, and pre‐ and post‐test counselling was provided for all. Participants were followed‐up after testing, and if reported reactive, were further supported for linkage to confirmatory testing and antiretroviral therapy (ART) initiation.

**Results:**

Among the 90,605 participants found eligible, 88,080 (97%) accepted an HIVST kit. Among the 87,976 who reported using an HIVST kit, 45,207 (51%) preferred a blood‐based kit, and 42,120 (48%) reported testing for the first time. For future testing, 77,064 (88%) reported preferring HIVST over other HIV testing methods. Among those who used the kit, 83,308 (95%) found the kit easy to use, and 83,237 (95%) reported that the test results were easy to interpret. Among those who preferred HIVST for future use, 52,136 (69%) were willing to pay for the kit, with 35,854 (69%) of those willing to pay less than US$ 1.20. Only one instance of social harm was reported, with a participant reporting suicidal tendencies due to discord with their partner.

Out of 328 participants (0.4%) who tested reactive with HIVST, 291 (89%) were linked to confirmatory testing; of these, 254 were confirmed HIV positive, and 216 (85%) successfully initiated ART.

**Conclusions:**

Overall, we report that nearly all participants were willing to accept HIVST, found the test kits easy to use and interpret, and about two‐thirds were willing to pay for HIVST. Given the high levels of acceptance and the ability to reach a large proportion of first‐time testers, HIVST in India could contribute to achieving the UNAIDS first 95 and ending the HIV epidemic.

## 1 INTRODUCTION

Substantial progress with access to HIV testing has been made, with 85% of people living with HIV (PLHIV) globally and 79% of estimated PLHIV in India knowing their HIV status in 2023 [1, 2]. However, a wider range of HIV testing options are needed to achieve the UNAIDS 95‐95‐95 targets. HIV self‐testing (HIVST) is an important tool to reach those who otherwise would not seek testing at healthcare facilities or community‐based testing sites [3].

Given the evidence available on the benefits of HIVST, the World Health Organization incorporated HIVST into its 2021 Consolidated Guidelines on HIV Prevention, Testing, Treatment, Service Delivery and Monitoring [4]. These guidelines recommend the distribution of HIVST kits through various community and facility‐based models. It also recommends private practitioners, pharmacies, workplaces and online delivery of HIVST kits. HIVST kit delivery through innovative distribution mechanisms has been shown to be feasible and acceptable [5, 6].

Considering these benefits associated with the introduction of HIVST kits, India is also taking steps towards adapting its HIV‐testing guidelines to include HIVST. Currently, HIVST kits are not available for purchase in the country, and there is no established HIVST policy. The National AIDS and STD Control Programme (NACP) Phase‐V (2021−2026) calls for the generation of evidence regarding implementation modalities for introducing HIVST in the country [7]. Studies from India report that HIVST is acceptable among key populations, such as men who have sex with men (MSM), transgender people (TG), female sex workers (FSWs) and truckers [8, 9, 10, 11, 12, 13, 14].

However, most of these studies were qualitative, and there is limited evidence available on models for the distribution of HIVST. To fill this evidence gap, we implemented a demonstration study to understand the acceptability, usability and willingness to pay for HIVST distributed through various distribution models and among diverse population groups in India. Here, we present results from the distribution of HIVST through three different models.

## 2 METHODS

### 2.1 Study design and setting

This cross‐sectional study was conducted across 50 districts in 14 states in India with a high prevalence of HIV and/or a high number of PLHIV [2]. In 2020, these states contributed over 55% of the new HIV cases in the country [2]. The study included states from all regions of the country and included both urban and rural areas. The complete list of models implemented by states and districts is provided in Table S1.

### 2.2 HIVST distribution models

HIVST kits were distributed in the study using five models. Three models focused on in‐person distribution and reaching key populations, their partners, clients and partners of PLHIV are presented here. These include the community‐based, PLHIV network‐led and private practitioners models. The other models implemented were workplace and virtual models, the results of which are yet to be published.

### 2.3 Community‐based model

The community‐based model was implemented in all 14 states. In this model, HIVST kits were distributed by study staff who were supported by community‐based organisations (CBOs) providing HIV testing services through targeted intervention or other community‐based testing methods. This model was designed to reach key populations (FSWs, MSM, TG and people who inject drugs [PWID]), their sexual and injecting partners, and clients, and partners of PLHIV. Bridge populations such as migrant workers and truckers were also reached. HIVST distribution in this model took place from September 2021 to June 2022.

**Figure 1:**
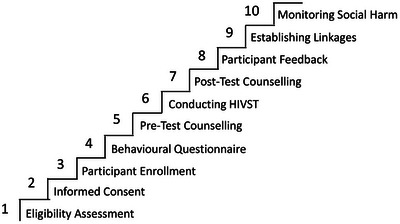
Study process followed in the STAR III study in India.

Study staff were recruited mainly from key population communities. All staff received two days in‐person training from the study investigators and master trainers in their local language. The training covered the basics of HIV transmission and treatment, HIV testing approaches and study procedures which included conducting the eligibility assessment, consent‐taking procedures, study definitions, pre‐and post‐test counselling, HIVST kit demonstrations, follow‐up protocol, social harms assessment and responding to any reported social harms, and data capture. The study process is shown in Figure 1.

The study staff conducted demand‐generation activities such as conducting camps and community information sessions at hotspots, CBO offices and other community‐based testing sites. Participants who met the eligibility criteria and provided written informed consent were enrolled in the study and offered to select between blood‐based (Mylan HIV self‐test, Atomo Diagnostics Pvt. Ltd, Australia or Insti HIV self‐test, bioLytical Lab., Canada) or oral‐fluid‐based (OraQuick HIV self‐test, OraSure Technologies, USA) HIVST kits and to test in an assisted or unassisted approach. Assisted testing was defined as testing in the physical presence of study staff. All participants were shown kit demonstration videos (available at https://www.youtube.com/@starhivselftesting3897) before selecting a kit. Pre‐ and post‐test counselling was conducted. Participants who opted for the unassisted approach could test at the distribution site or take the test kit to test later. Participants who took the test kits with them were followed up on days 3, 5 and 7 before being declared lost to follow‐up. Participants were followed‐up by phone or in‐person. Any participant who requested additional time for testing was further followed‐up.

All participants with an HIV reactive self‐test were offered accompanied referral to the nearest public Integrated Counselling and Testing Centre (ICTC) for confirmatory testing and to an antiretroviral therapy (ART) centre for ART initiation for those who were confirmed HIV positive.

All participants were followed up by phone or in person after 7 days of testing to check for social harm and those who reported it were offered appropriate support.

### 2.4 PLHIV network‐led model

This model aimed to reach the partners of PLHIV in six districts of one state (Table S1). It was implemented by a CBO formed by PLHIV that runs a peer‐led integrated health centre and community pharmacy offering positive prevention counselling, HIV testing for spouses/partners and children of PLHIV, subsidised ART, pre‐exposure prophylaxis (PrEP) and post‐exposure prophylaxis (PEP). The distribution of kits through this model occurred between January and June 2022.

The staff, who were PLHIV, received standard training and followed study procedures as described in the community‐based model. Demand generation was conducted through physical and virtual modes with the registered PLHIV, their partners and any client seeking HIV testing services at one of the service delivery sites.

### 2.5 Private practitioners model

The private practitioners model was implemented in nine districts from five states, selected based on administrative approvals and operational feasibility (Table S1). Private practitioners who provided sexually transmitted infection (STI) services, tuberculosis clinics, dermatologists and family physicians/general practitioners (including allopathic, ayurvedic and homoeopathic doctors) providing HIV and STI services were mapped. A total of 700 private practitioners and labs were mapped and approached. Among these, 7.1% (50) practitioners agreed to participate in the study. The availability of in‐house laboratory services and the perceived lengthiness of the study procedures were the main reasons reported for low participation. A two‐day training on HIVST and the study procedures were conducted for the private practitioner or their support staff (nurses and lab technicians). A study staff was assigned to support study activities at the clinics. Study staff would be present at the clinic on days and times agreed with the practitioner. For high‐burden clinics, monetary incentives were given to clinic staff to act as the study focal person. Follow‐up visits were conducted by the study staff every two weeks to discuss challenges and to arrive at appropriate solutions.

Posters and takeaway leaflets on HIVST were kept inside the clinics. Clients willing to receive HIVST were asked to approach the designated clinic staff. The practitioners also referred their clients to the study staff. The study staff guided all potential participants through the study processes as described in the community‐based model. Kit distribution through this model occurred between December 2021 and June 2022.

### 2.6 Study participants

The study included participants who were 18 years of age or above and identified with one of the following groups—member of a key population group (FSW, MSM, TG, PWID), partner or client of key population, partner of PLHIV, referred from identified private practitioners or individuals self‐identified at high‐risk of HIV. HIV‐positive persons, those on PrEP or PEP and pregnant women were not included in the study. While both the blood and oral‐fluid‐based HIVST kits were offered to all the participants, those who self‐reported ever testing positive for hepatitis B or C were offered only the blood‐based HIVST kit.

### 2.7 Data collection and analysis

A structured questionnaire was used to collect data. Data were collected directly into a mobile phone‐based web application. Staff were trained to collect data in real‐time while completing the study procedures. In locations with a poor network, data were collected using hard‐copy forms and then entered into the online tool once the network was available.

The primary outcomes measured were the acceptability of HIVST for current and future testing, usability of the HIVST kits (including ease of use and result interpretation) and willingness to pay (Table 1). To understand participants’ willingness to pay, after the testing process, they were asked for a range within which they would be willing to pay for an HIVST kit. To determine the factors associated with the primary outcomes, we first calculated the unadjusted odds ratios and 95% confidence intervals. Variables with *p*‐value<0.20 were selected for multiple regression. The variables population group and gender were found to be collinear, hence, only population group was used for further analysis. Using these variables, directed acyclic graphs were constructed (Figures S1−S3) to identify potential confounders for each outcome‐factor association. Confounders to be adjusted using the multiple logistic regression were identified by comparing the −2 log‐likelihood ratios of the models with and without the confounding variables, separately for each exposure variable [15]. Data were analysed using SPSS (Version 20, 2011; IBM Inc., Chicago, IL, USA) and Stata (Version 16, StataCorp LLC, Texas, USA).

**Table 1:** Outcome definitions of primary outcomes for the STAR III study in India

Outcome	Definition
Acceptance of HIVST	The proportion of participants who accepted to receive HIVST among those eligible
Acceptance of HIVST for future testing	Among those who used the HIVST kit, the proportion of participants who indicated willingness to use HIVST for future testing
Ease of using the HIVST kit	Among those who used the HIVST kit, the proportion of participants who reported finding the test kit easy or very easy to use
Ease of result interpretation	Among those who used the HIVST kit, the proportion of participants who reported finding the test kit easy or very easy to interpret
Willingness to pay	Among those who preferred HIVST for future testing, the proportion of those who were willing to pay

### 2.8 Ethics

The study was approved by the ethics committees of The Humsafar Trust, Mumbai, India (HST‐IRB‐49‐10/2020), and WCG IRB, USA (20212973). The study also received a research determination from the Scientific Integrity Branch of the Division of Global HIV and TB, CDC, Atlanta. Written informed consent was obtained from all participants.

## 3 RESULTS

**Figure 2:**
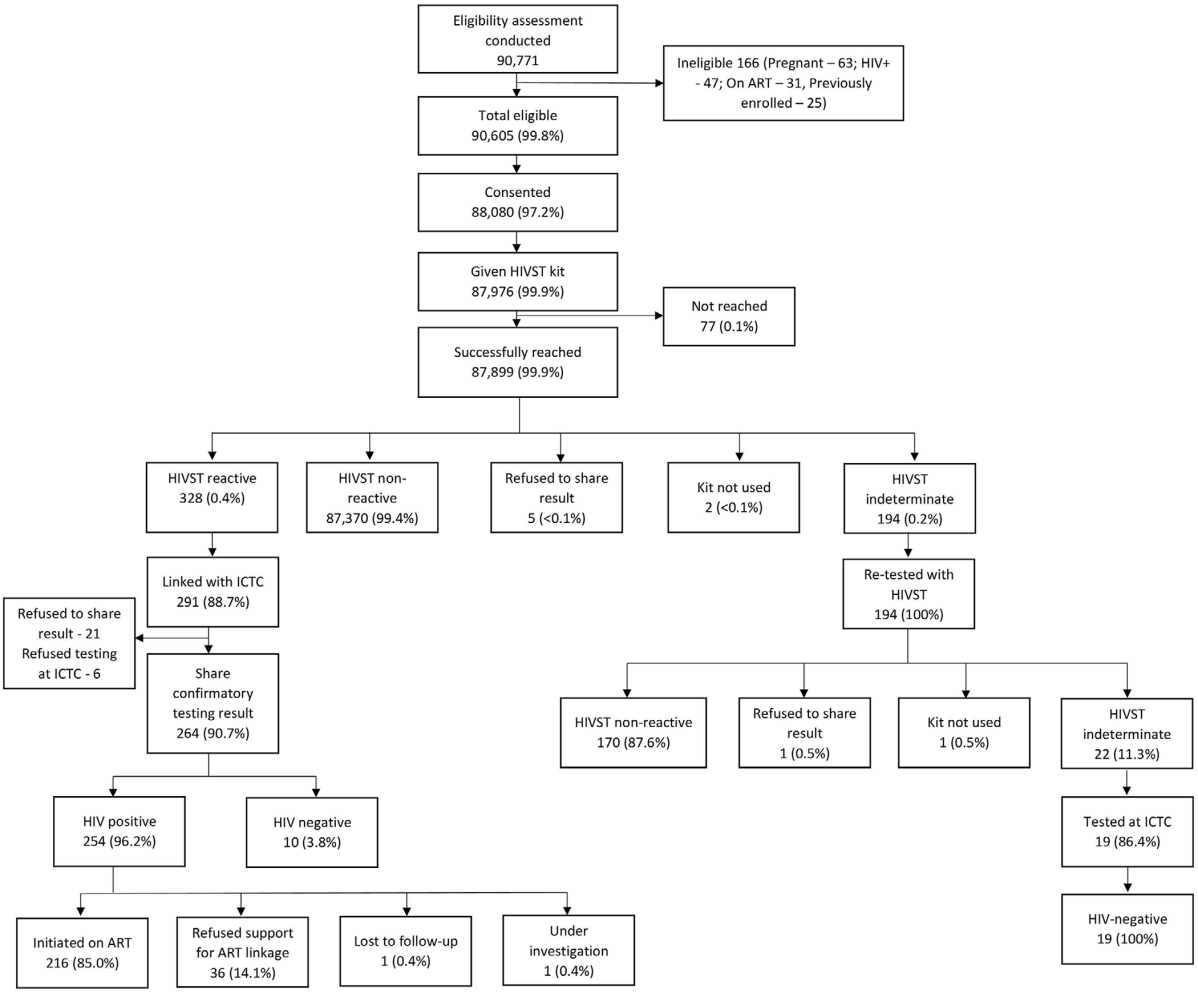
Study cascade for the STAR III study in India.

A total of 90,771 people agreed to check their eligibility, among whom 90,605 (99.8%) were found eligible (Figure 2). Among those eligible, 88,080 (97%) consented to accept an HIVST kit, while 87,976 (99.9%) received an HIVST kit. Thus, 97% (87,976/90,605) of those eligible accepted an HIVST kit. Most of those who received an HIVST kit were recruited through the community‐based model (79,324/87,976; 90%), followed by the private practitioners model (6984; 8%) and the PLHIV network‐led model (1668; 2%) (Table 2).

**Table 2:** Socio‐demographic characteristics of the population enrolled in the STAR III study in India

	Community‐based model	PLHIV network‐led model	Private practitioners model	Total
Total	79,324 (100)	1668 (100)	6984 (100)	87,976 (100)
**Place of recruitment**				
CBO/NGO/other community‐based testing sites	47,161 (59)	170 (10)	436 (6)	47,767 (54)
Home visit	21,889 (28)	992 (59)	34 (0.5)	22,915 (26)
Private clinic/pharmacy	233 (0.3)	482 (30)	6510 (93)	7225 (8)
Virtual outreach	560 (0.7)	0 (0)	0 (0)	560 (0.6)
Other	9481 (12)	24 (1)	4 (0.1)	9509 (11)
Median age (IQR)	30 (25−35)	32 (24−42)	32 (28−38)	30 (26−35)
**Age**				
18−24 years	16,151 (20)	425 (25)	761 (11)	17,337 (20)
25−34 years	41,078 (52)	493 (30)	3607 (51)	45,178 (51)
35−44 years	18,231 (23)	393 (24)	2196 (31)	20,820 (24)
>44 years	3864 (5)	357 (21)	420 (6)	4641 (5)
**Gender**				
Male	51,479 (65)	815 (49)	5633 (81)	57,927 (66)
Female	23,008 (29)	850 (51)	1349 (19)	25,207 (29)
Transgender	4765 (6)	3 (0.2)	2 (<0.1)	4770 (5)
Other	72 (0.1)	0	0	72 (0.1)
**Population group**				
FSW	19,513 (25)	24 (1)	295 (4)	19,832 (22)
MSM	15,180 (19)	26 (2)	36 (0.5)	15,242 (17)
TG	4765 (6)	3 (0.2)	2 (<0.1)	4770 (5)
PWID	5528 (7)	3 (0.2)	2 (<0.1)	5533 (6)
Partner/client of key population	10,934 (14)	24 (1)	41 (0.6)	10,999 (12)
Partner of PLHIV	1855 (2)	537 (32)	3 (<0.1)	2395 (3)
Family member of PLHIV	0	340 (20)	0	340 (0.4)
Private practitioner/other referrals	353 (0.4)	91 (5)	6111 (87)	6555 (7)
Self‐identified high‐risk individuals	21,199 (27)	620 (37)	494 (7)	22,313 (25)
**Education (*n* = 87,493)**				
No formal education	5382 (7)	123 (7)	136 (2)	5641 (6)
Primary	30,128 (38)	447 (27)	2469 (35)	33,044 (38)
High school	36,451 (46)	987 (59)	3605 (52)	41,043 (47)
Higher education	6888 (9)	103 (6)	774 (11)	7765 (9)
**HIV risk perception (*n* = 86,599)**				
Low risk	22,218 (28)	833 (51)	1687 (24)	24,738 (29)
Medium risk	33,634 (43)	413 (25)	2953 (43)	37,000 (43)
High risk	11,953 (15)	294 (18)	839 (12)	13,086 (15)
Don't know	10,271 (13)	97 (6)	1407 (20)	11,775 (14)
**Last HIV test (*n* = 87,974)**				
Never tested	35,740 (45)	1144 (69)	5236 (75)	42,120 (48)
0−12 months	26,874 (34)	176 (11)	706 (10)	27,756 (32)
More than 12 months	11,681 (15)	290 (17)	493 (7)	12,464 (14)
Time since last test not known	5027 (6)	58 (3)	549 (8)	5634 (6)
**Kit preference**				
Blood‐based	39,967 (50)	713 (43)	4526 (65)	45,206 (51)
Oral‐fluid based	39,357 (50)	955 (57)	2458 (35)	42,770 (49)

Abbreviations: CBO, community‐based organization; FSW, female sex worker; IQR, inter quartile range; MSM, men who have sex with men; NGO, non‐governmental organisation; PWID, people who inject drugs; TG, transgender people.

### 3.1 Population characteristics

Participants median age was 30 years (IQR: 26−35 years) (Table 2). The median age was higher for participants recruited through the PLHIV network‐led (32 years; IQR: 24−42) and private practitioners (32 years; IQR: 28−38) models. Overall, 65.8% of participants were men, the proportion of women in the PLHIV network‐led model was relatively higher (51%). TG were mainly recruited through the community‐based model. Based on the recruitment strategies, the community‐based model enrolled mostly FSW, MSM and other self‐identified high‐risk individuals, while the PLHIV network‐based model reached partners and family members of PLHIV.

### 3.2 First‐time testers

The study reached 42,120 (48%) first‐time testers (Table 3). The proportion of first‐time testers was 75% in the private practitioners model and 69% in the PLHIV network‐led model. A higher proportion of men (55%) and younger individuals (54% among those between 18 and 24 years) were first‐time HIV testers. Almost all family members of PLHIV (98%) reported testing for the first time. The proportion of first‐time testers was also high among private practitioner referrals (76%), partners/clients of key population groups (68%) and MSM (43%).

**Table 3:** Time since last HIV test and HIVST kit type preference for participants who accepted an HIVST kit in the STAR III study in India

	Time since last HIV test				HIVST kit type preference			
	Total	Never tested	0−12 months	12+ months	Unknown time of last HIV test	Total	Oral fluid‐based	Blood‐based
Total	87,974 (100)	42,120 (48)	27,756 (32)	12,464 (14)	5634 (6)	87,976 (100)	42,769 (49)	45,207 (51)
**Model**								
Community‐based model	79,322 (100)	35,740 (45)	26,874 (34)	11,681 (15)	5027 (6)	79,324 (100)	39,341 (50)	39,983 (50)
PLHIV network‐led model	1668 (100)	1144 (69)	176 (11)	290 (17)	58 (3)	1668 (100)	959 (57)	709 (42)
Private practitioners’ model	6984 (100)	5236 (75)	706 (10)	493 (7)	549 (8)	6984 (100)	2469 (35)	4515 (65)
**Age**								
18−24 years	17,337 (100)	9440 (54)	4700 (27)	1927 (11)	1270 (7)	17,337 (100)	8606 (50)	8731 (50)
25−34 years	45,117 (100)	21,103 (47)	14,846 (33)	6903 (15)	2325 (5)	45,178 (100)	22,151 (49)	23,027 (51)
35−44 years	20,819 (100)	9293 (45)	6903 (33)	2979 (14)	1644 (8)	20,820 (100)	9935 (48)	10,885 (52)
>44 years	4641 (100)	2284 (49)	1307 (28)	655 (14)	395 (8)	4641 (100)	2077 (45)	2564 (55)
**Gender**								
Male	57,926 (100)	32,153 (55)	13,853 (24)	7207 (12)	4713 (8)	57,927 (100)	27,023 (47)	30,904 (53)
Female	25,206 (100)	8293 (33)	12,125 (48)	4032 (16)	756 (3)	25,207 (100)	13,112 (52)	12,095 (48)
Transgender	4770 (100)	1635 (34)	1775 (37)	1223 (26)	137 (3)	4770 (100)	2583 (54)	2187 (46)
Other	72 (100)	39 (54)	3 (4)	2 (3)	28 (39)	72 (100)	51 (71)	21 (29)
**Population group**								
FSW	19,831 (100)	5168 (26)	11,261 (57)	2921 (15)	481 (2)	19,832 (100)	10,419 (52)	9413 (47)
MSM	15,241 (100)	6558 (43)	5409 (35)	2280 (15)	994 (6)	15,242 (100)	8551 (56)	6691 (44)
TG	4764 (100)	1633 (34)	1773 (6)	1221 (26)	137 (3)	4770 (100)	2583 (54)	2187 (46)
PWID	5534 (100)	1366 (25)	3044 (55)	805 (14)	319 (6)	5533 (100)	1875 (34)	3658 (66)
Partner/client of key population	11,001 (100)	7471 (68)	1410 (13)	1173 (11)	947 (9)	10,999 (100)	4644 (42)	6355 (58)
Partner of PLHIV	2392 (100)	800 (33)	769 (32)	727 (30)	96 (4)	2392 (100)	1273 (53)	1119 (47)
Family member of PLHIV	340 (100)	334 (98)	1 (0.3)	0 (0)	5 (1)	340 (100)	161 (47)	179 (53)
Private practitioner/other referrals	6555 (100)	4992 (76)	527 (8)	464 (7)	572 (9)	6555 (100)	2524 (38)	4031 (61)
Self‐identified high‐risk individuals	22,313 (100)	13,798 (62)	3561 (16)	2871 (13)	2083 (9)	22,313 (100)	10,739 (48)	11,574 (52)

Abbreviations: FSW, female sex worker; MSM, men who have sex with men; PWID, people who inject drugs; TG, transgender people.

### 3.3 HIVST testing cascade

Among the 87,976 participants who received an HIVST kit, 87,899 (99.9%) were reached for follow‐up (Figure 2). Five participants (<0.01%) declined to share their test results. One hundred ninety‐four (0.2%) tested indeterminate on the first HIVST kit, and were retested; 22 (11%) tested indeterminate the second time and were offered linkage to the nearest ICTC for retesting. All 22 participants had used a blood‐based kit and used the assisted testing approach. Of these, 19 (86%) participants were tested at ICTC and all tested HIV negative.

A total of 328 (0.4%) participants received a reactive HIVST result. Among these, 291 (89%) were linked for confirmatory testing at a public ICTC. Among 254 who were confirmed HIV positive, 216 (85%) were linked to ART. A total of 10 (4%) tested negative in confirmatory testing. Of the 328 who tested HIVST reactive, 175 (53%) reported testing for the first time.

### 3.4 Test kit type preference

Among the 87,976 participants, a slightly higher preference was seen for blood‐based kits with 45,207 (51%) participants opting for them (Table 3). However, females (12,112; 52%), TG (2583; 54%) and MSM (8551; 56%) selected the oral‐fluid‐based test kit. A higher proportion of people referred by private practitioners preferred the blood‐based kit (4031; 61%).

### 3.5 Acceptance of HIVST for future testing

Among the 87,976 participants who tested with an HIVST kit, 77,064 (88%) reported willingness to use HIVST over other HIV testing services in the future (Table 4). Partner/family members of PLHIV (OR: 3.668; 95 CI: 2.995−4.492) and TG (OR: 1.564; 95 CI: 1.400−1.747) had higher odds of preferring HIVST over other testing methods. People who considered themselves at high risk of HIV (aOR: 0.600; 95 CI: 0.561−0.643) and those referred by private practitioners (OR: 0.582; 95 CI: 0.541−0.626) had lower odds for using HIVST in the future compared to those who were unaware of their HIV risk and those who self‐identified themselves at high risk, respectively.

**Table 4:** Acceptance of HIVST for future testing, ease of using an HIVST kit and ease of interpreting the result among participants of the STAR III study in India

		**Acceptance for future use**					**Ease of using the kit**					**Ease of interpretation**				
	**Total**	**Yes *n* (%)**	**OR**	**95 CI**	**aOR**	**95 CI**	**Easy *n* (%)**	**OR**	**95 CI**	**aOR**	**95 CI**	**Easy *n* (%)**	**OR**	**95 CI**	**aOR**	**95 CI**
Total	87,976	77,064 (88)					83,308 (95)					83,273 (95)				
**Age**																
18−24 years	17,337	15,639 (90)	Ref				16,172 (93)	Ref				16,136 (93)	Ref			
25−34 years	45,178	39,400 (87)	0.74	0.699−0.784			42,768 (95)	1.278	1.189−1.374			42,787 (95)	1.332	1.240−1.431		
35−44 years	20,820	17,834 (86)	0.648	0.609−0.691			19,870 (95)	1.507	1.380−1.646			19,877 (95)	1.569	1.437−1.713		
>44 years	4641	4191 (90)	1.011	0.906−1.128			4498 (97)	2.266	1.899−2.704			4473 (96)	1.982	1.681−2.337		
**Gender**																
Male	57,927	51,418 (89)	Ref				54,352 (94)	Ref				54,243 (94)	Ref			
Female	25,207	21,228 (84)	0.675	0.647−0.705			24,358 (97)	1.887	1.748−2.037			24,521 (97)	2.428	2.235−2.637		
Transgender	4770	4376 (92)	1.406	1.264−1.564			4536 (95)	1.275	1.113−1.460			4452 (93)	0.951	0.845−1.071		
Prefer not to disclose	72	42 (58)	0.177	0.111−0.283			62 (86)	0.408	0.209−0.796			57 (79)	0.258	0.146−0.456		
**Population group**																
Self‐identified high‐risk individuals	22,313	19,562 (88)	Ref				20,904 (94)	Ref				20,885 (94)	Ref			
FSW	19,832	16,546 (83)	0.708	0.670−0.748			19,193 (97)	2.025	1.840−2.227			19,289 (97)	2.429	2.196−2.686		
MSM	15,242	13,669 (90)	1.222	1.144−1.305			14,281 (94)	1.002	0.920−1.090			14,277 (94)	1.012	0.930−1.101		
TG	4764	4371 (92)	1.564	1.400−1.747			4531 (95)	1.311	1.137−1.511			4450 (93)	0.969	0.854−1.100		
PWID	5534	4814 (87)	0.94	0.861−1.027			4959 (90)	0.581	0.525−0.644			4922 (89)	0.55	0.498−0.608		
Partner/client of key population	11,001	10,188 (93)	1.762	1.624−1.913			10,406 (95)	1.179	1.068−1.301			10,394 (94)	1.171	1.062−1.291		
Partner/family member of PLHIV	2735	2634 (96)	3.668	2.995−4.492			2658 (97)	2.327	1.843−2.937			2678 (98)	3.212	2.458−4.199		
Private practitioner/other referrals	6555	5280 (80)	0.582	0.541−0.626			6376 (97)	2.401	2.050−2.812			6378 (97)	2.464	2.102−2.887		
**Education (*n* = 87,493)**																
No formal education	5641	4945 (88)	Ref				5497 (97)	Ref		Ref^a^		5457 (97)	Ref		Ref	
Primary	33,044	28,927 (87)	0.989	0.908−1.078			31,747 (96)	0.641	0.539−0.763	0.653	0.548−0.778	31,694 (96)	0.792	0.677−0.926	0.802	0.686−0.939
High school	41,043	36,025 (88)	1.01	0.928−1.100			38,435 (94)	0.386	0.326−0.458	0.408	0.344−0.484	38,546 (94)	0.521	0.447−0.606	0.548	0.471−0.639
Higher education	7765	6825 (88)	1.022	0.920−1.135			7262 (93)	0.378	0.313−0.457	0.405	0.335−0.490	7250 (93)	0.475	0.400−0.564	0.506	0.426−0.602
**HIV risk perception (*n* = 86,599)**																
Don't know	11,775	10,036 (85)	Ref		Ref^b^		10,856 (92)	Ref		Ref^c^		10,778 (91)	Ref		Ref^c^	
Low risk	24,738	23,089 (93)	2.426	2.259−2.606	2.321	2.159−2.495	23,576 (95)	1.718	1.571−1.878	1.764	1.609−1.933	23,521 (95)	1.788	1.639−1.905	1.87	1.710−2.044
Medium risk	37,000	32,602 (88)	1.284	1.210−1.364	1.247	1.173−1.325	35,050 (95)	1.522	1.403−1.651	1.483	1.364−1.612	35,117 (95)	1.725	1.593−1.868	1.744	1.606−1.894
High risk	13,086	10,253 (78)	0.627	0.587−0.670	0.6	0.561−0.643	12,591 (96)	2.153	1.925−2.409	2.028	1.805−2.279	12,641 (97)	2.628	2.343−2.947	2.469	2.192−2.781
**Last HIV test (*n* = 87,974)**																
Never tested	42,120	37,427 (89)	Ref		Ref^d^		39,704 (94)	Ref		Ref^e^		39,681 (94)	Ref		Ref^e^	
0−12 months	27,756	23,460 (84)	0.685	0.655−0.716	0.717	0.680−0.755	26,584 (96)	1.38	1.285−1.483	1.392	1.284−1.509	26,622 (96)	1.443	1.343−1.551	1.429	1.317−1.551
More than 12 months	12,464	11,202 (90)	1.113	1.042−1.189	1.137	1.061−1.218	11,829 (95)	1.134	1.036−1.240	1.183	1.076−1.302	11,894 (95)	1.283	1.168−1.408	1.349	1.220−1.490
Time since last test not known	5634	4974 (88)	0.945	0.867−1.031	1.01	0.919−1.109	5190 (92)	0.711	0.640−0.790	0.798	0.713−0.892	5075 (90)	0.558	0.507−0.615	0.627	0.565−0.695
**Kit used**																
Oral‐fluid	42,769	37,563 (88)	Ref		Ref^f^		41,106 (96)	Ref		Ref^g^		40,831 (95)	Ref		Ref^g^	
Blood‐based	45,207	39,501 (87)	0.959	0.922−0.999	0.946	0.907−0.986	42,202 (93)	0.568	0.534−0.604	0.555	0.521−0.591	42,442 (94)	0.729	0.686−0.773	0.725	0.682−0.772

^a^ Adjusted for age.

^b^ Adjusted for age and population group.

^c^ Adjusted for age, education and population group.

^d^ Adjusted for age, population group and HIV risk perception.

^e^ Adjusted for age, education, HIV risk perception and population group.

^f^ Adjusted for age, HIV risk perception and last HIV test.

^g^ Adjusted for age, population group, HIV risk perception and last HIV test.

The major reasons that participants reported for selecting HIVST over other testing methods were privacy (84%; 65,011/77,064), getting quick results with HIVST (81%, 62,371/77,064), convenience (44%; 34,157/77,064) and ease of administration (40%, 31,077/77,064). Participants reported that the possibility of interpreting the result incorrectly (8%; 6172/77,064), getting a false negative or positive result (8%; 5959/77,064) and the loss of opportunity to interact with a healthcare provider and counselling (6%; 4635/77,064) were the concerns with selecting HIVST over healthcare provider delivered HIV testing.

### 3.6 Ease of using the kit and interpreting the result

Overall, 83,380 (95%) and 83,237 (95%) participants reported that they found the test kits easy to use and to interpret the result, respectively (Table 4). Participants with higher education had lower odds of finding the kits easy to use (aOR: 0.405; 95 CI: 0.335−0.490) and interpret (aOR: 0.506; 95 CI: 0.426−0.602) than those with no formal education. Also, participants using the blood‐based kits had lower odds of finding the kits as easy to use (aOR: 0.555; 95 CI: 0.521−0.591) and interpret (aOR: 0.725; 95 CI: 0.682−0.772) compared to those using oral‐fluid based test kits. Higher risk perception levels, recent HIV testing and use of oral fluid kit were associated with greater ease of interpretation, while low and medium HIV risk perceptions and use of oral‐fluid kit increased the likelihood of future use.

### 3.7 Willingness to pay

Among the 75,206 participants who preferred HIVST for future testing, 52,136 (69%) reported willingness to pay for the test kit in the future (Table 5). A large proportion of TG (88%) and MSM (72%) reported willingness to pay for the kit. A total of 35,854 (48%) participants were willing to pay less than US$ 1.2 (INR 100) to purchase the kit, 13,857 (18%) reported a willingness to pay between US$ 1.2 and 3 (INR 101−250), and only 2425 (3%) reported a willingness to pay more than US$ 3 (INR 250). Just over one‐third of TG (40%) were willing to pay between US$ 1.2 and 3 (INR 101−250) for the HIV self‐test kits.

**Table 5:** Willingness to pay for HIV self‐testing among participants of the STAR III study in India

	Not willing to pay	Less than USD 1.2 (INR 100)	Between USD 1.2 and USD 3 (INR 101−250)	More than USD 3 (INR 250)	Total
Total	23,070 (1)	35,854 (48)	13,857 (18)	2425 (3)	75,206
**Model**					
Community‐based model	20,252 (29)	33,003 (48)	13,023 (19)	2238 (3)	68,561
PLHIV network‐led model	420 (26)	905 (56)	255 (16)	31 (2)	1611
Private practitioners model	2398 (48)	1946 (39)	579 (11)	111 (2)	5034
**Age**					
18−24 years	4665 (31)	7206 (48)	2759 (18)	472 (3)	15,092
25−34 years	11,845 (31)	18,041 (47)	7206 (19)	1320 (3)	38,412
35−44 years	5314 (30)	8677 (49)	3121 (18)	449 (2)	17,561
>44 years	1256 (30)	1930 (46)	771 (17)	184 (4)	4141
**Gender**					
Male	15,314 (30)	25,383 (51)	8197 (16)	1300 (3)	50,194
Female	7217 (35)	8566 (41)	3909 (19)	939 (4)	20,631
Transgender	537 (12)	1887 (43)	1733 (40)	182 (4)	4339
Other	2 (5)	18 (25)	18 (43)	4 (9)	42
**Population group**					
FSW	5486 (34)	6513 (41)	3220 (20)	786 (5)	16,005
MSM	3671 (28)	6812 (51)	2256 (17)	507 (4)	13,246
TG	537 (12)	1887 (43)	1733 (40)	182 (4)	4339
PWID	1516 (33)	2033 (44)	1009 (22)	57 (1)	4615
Partner/client of key population	3896 (40)	3895 (40)	1813 (18)	200 (2)	9804
Partner of PLHIV	547 (24)	1180 (52)	461 (20)	91 (4)	2279
Family member of PLHIV	257 (78)	72 (22)	0	0	329
Private practitioner/other referrals	2472 (47)	2049 (39)	628 (12)	115 (2)	5264
Self‐identified high‐risk individuals	4688 (24)	11,413 (59)	2737 (14)	487 (2)	19,325
**Preferred HIVST kit for future use**					
Blood‐based	11,650 (33)	16,642 (48)	5654 (16)	949 (3)	34,895
Oral fluid‐based	11,298 (28)	19,164 (48)	8182 (20)	1469 (4)	40,113

Abbreviations: FSW, female sex worker; MSM, men who have sex with men; PWID, people who inject drugs; TG, transgender people.

### 3.8 Social harms

One participant identifying as MSM, with a history of depression and suicidal thoughts, who tested HIV positive reported having conflict with his partner and suicidal thoughts. That participant was linked with psychological counselling.

## 4 DISCUSSION

HIVST kits distributed through various community‐based organisations and private providers was seen to be highly acceptable among a wide range of key populations and other high‐risk groups in India. Over 97% of those eligible accepted HIVST kits and almost 9 out of 10 participants reported preferring to receive HIVST over provider‐delivered testing methods in the future. Several studies from India have previously reported that HIVST was acceptable among key population groups in the country [8–12, 14]. While most of these studies were either qualitative or had a limited sample size, this study, for the first time in the country, demonstrated the high acceptability of HIVST empirically through different distribution models. HIVST distribution in private clinics/hospitals could be encouraged by offering training to clinic staff to help integrate HIVST distribution into the clinic workflows and displaying educational materials on HIVST in the clinics.

As of 2021−22, the NACP reports coverage of over 90% of the estimated number of people in the key population groups [2]. However, many study participants from these groups reported testing for the first time. We also found a large number of first‐time testers among the partners and clients of key populations. This proportion was as high as reported from Vietnam and China and higher than seen in other settings such as Malawi, Zambia and Zimbabwe, underscoring its value in expanding reach and improving HIV diagnosis as India advances towards epidemic control goals [16, 17, 18].

ART initiation in this study at 85% was only slightly lower than the national linkage of 92% from public ICTCs to ART centres, however, importantly diagnosed people who never before tested [19]. Previous studies among FSWs, pregnant women and truckers in India reported a preference for oral‐fluid test kits over blood‐based [9, 10, 11, 12]. However, in the present study, we found that an almost equal proportion of participants preferred oral‐fluid and blood‐based test kits.

Along with high acceptance, this study also found high ease of use and ease of interpretation for both, oral‐fluid and blood‐based test kits, irrespective of the education level of the participants. This finding is in concurrence with other studies around the world [5, 6]. Given the high acceptability and usability of both oral‐fluid and blood‐based HIVST kits, it will be imperative to have both kit types made available in the country.

Seven out of 10 participants who preferred HIVST kits for future testing reported willingness to pay at least some amount for them. This proportion is similar to studies from low‐ and middle‐income countries that report willingness to pay between 65% and 92% [20, 21, 22]. Among those who were willing to pay, 69% were willing to pay up to US$ 1.2 or lower, and 26.6% were willing to pay up to US$ 3. This amount is similar to that reported by other studies from low‐ and middle‐income countries [20, 21, 22, 23, 24]. Given the mismatch between the current costs of the test kits (US$ 7−US$ 12 in the private sector) and the price most participants are willing to pay, it will be imperative to continue with market interventions to reduce the costs of test kits for end users [25]. Given the ability to reach a large proportion of first‐time testers and high levels of acceptance and usability of HIVST, there is value in exploring making HIVST available from the public sector.

We found extremely low levels of reported social harms in our study, with only one participant reporting suicidal tendencies after testing HIV positive. Further investigation revealed that the suicidal tendency was due to extraneous factors and, therefore, was not associated with the use of HIVST. These findings are in line with the international literature that report a very low level of social harms associated with the use of HIVST [6].

Based on overall outcomes, TG reported a high level of acceptance, usability and willingness to pay for the use of HIVST kits. This might be due to the inherent confidentiality and privacy that HIVST has to offer, thus sparing them from the discrimination they might face at healthcare facilities [26].

This study is among the first that provides evidence on the distribution of HIVST through different service delivery models among a wide range of population groups across 14 states and 50 districts in India. This study was conducted at existing HIV service sites and thus represents real‐world scenarios in the country. The COVID‐19 pandemic, despite leading to delays in obtaining regulatory approvals for importing HIVST kits, may not have significantly impacted the outcome. However, the study had some limitations. The study sampling frame was not designed to be nationally representative of key populations and their sexual/injecting partners or clients. It did not include pregnant women or private sector pharmacies nor allowed for secondary distribution. Future studies can look at the feasibility of using secondary distribution to reach population groups such as partners of MSM and FSWs that might be even more difficult to reach. As data were collected face‐to‐face, the possibility of desirability bias cannot be ruled out.

## 5 CONCLUSIONS

Overall, we found that nearly all participants were willing to accept HIVST, found the test kits easy to use and interpret, and about two‐thirds were willing to pay for HIVST distributed through the community‐based, PLHIV network‐led and private practitioners models in India, with good linkage to confirmatory testing and ART initiation. Given the high levels of acceptance and the ability to reach a large proportion of first‐time testers combined with the provision of comprehensive prevention solutions, HIVST in India could contribute to achieving the UNAIDS first 95 and ending the HIV epidemic.

## COMPETING INTERESTS

The authors declared no competing interests.

## AUTHORS’ CONTRIBUTIONS

CL, KM, AD, AH, VC, VV, CD, JVRPR, DCSR and KG have made contributions to the conception. CL, KM, AD, AH, MS, MP, VC, SG, TB and DCSR designed the work. CL, KM, SM, MS, MD, MM, GSS, MP, VJ, VRP, MP, CU, SC, SU and MN led the acquisition of data. CL, KM, AD, AH, VR, YB, JC, VC, SG, TB and DCSR conducted the analysis and interpretation of data. CL has drafted the work. KM, AD, AH, MS, SM, MS, MD, MM, GSS, MP, VJ, VRP, VR, YB, JC, MP, CU, SC, SU, MN, VC, SG, TB, VV, CD, JVRPR, DCSR and KG substantively reviewed and revised it. All authors have approved the submitted version.

## FUNDING

This study was conducted under the STAR III Initiative grant from Unitaid and led by Population Services International (PSI). The HIVST kits used in the study were supplied to the Indian investigators under the UNITAID STAR‐III grant, with necessary licenses obtained from the Central Drugs Standard Control Organisation (CDSCO) under the Directorate General of Health Services, Ministry of Health & Family Welfare, Government of India to ensure compliance with Indian regulations.

## DISCLAIMER

The findings and conclusions in this study are those of the authors and do not necessarily represent the official position of the funding agencies.

## Supporting information


**Table S1**: State and district‐wise HIVST distribution models implemented under the STAR III study in India
**Table S2**: Questions, responses and dichotomization of the primary outcomes
**Figure S1**: Directed acyclic graph (DAG) of ease of interpreting the HIV self‐test result.
**Figure S2**: Directed acyclic graph (DAG) of ease of use of HIV self‐testing kit.
**Figure S3**: Directed acyclic graph (DAG) of acceptance of HIV self‐testing for next use.

## Data Availability

The datasets analysed during the current study are available from the corresponding author on reasonable request and after approval from the ethics committee.
