# Supplementary material for: Acceptability, usability, and willingness to pay for HIV self‐test kits distributed through community‐based, PLHIV network‐led and private practitioners models in India: Results from the STAR III Initiative
Source: J Int AIDS Soc. 2024 Aug 8;27(8):e26348. doi: 10.1002/jia2.26348 (PMC11310287; doi:10.1002/jia2.26348)

Supplementary materials

Supplementary tables

Supplementary Table S1: State and district-wise HIVST distribution models implemented under the STAR III study in India

Supplementary Table S2: Questions, responses and dichotomisation of the primary outcomes

Supplementary figures

Supplementary Figure S1: Directed acyclic graph (DAG) of ease of interpreting the HIV self-test result.

Supplementary Figure S2: Directed acyclic graph (DAG) of ease of use of HIV self-testing kit.

Supplementary Figure S3: Directed acyclic graph (DAG) of acceptance of HIV self-testing for next use.

Supplementary Table S1: State and district-wise HIVST distribution models implemented under the STAR III study in India

State	District	Models implemented
ANDHRA PRADESH	EAST GODAVARI	Community based model
	GUNTUR	Community based model
	KRISHNA	Community based model
	KURNOOL	Community based model
	VISAKHAPATNAM	Community based model
	GUNTUR	Community based model
DELHI	DELHI	Community based model
GUJARAT	AHMEDABAD	Community based model<br>Private practitioners' model
	VADODARA	Community based model<br>Private practitioners' model
KARNATAKA	BENGALURU	Community based model<br>Private practitioners' model
	MYSURU	Community based model
MADHYA PRADESH	BHOPAL	Community based model
	INDORE	Community based model
	JABALPUR	Community based model
	JHABUA	Community based model
	NEEMUCH	Community based model
	UJJAIN	Community based model
MAHARASHTRA	AHMEDNAGAR	Community based model<br>PLHIV network led model
	CHANDRAPUR	Community based model<br>PLHIV network led model
	JALGAON	Community based model<br>PLHIV network led model
	MUMBAI	Community based model
	MUMBAI SUBURBAN	Community based model
	NAGPUR	Community based model<br>PLHIV network led model
	NASHIK	Community based model<br>PLHIV network led model
	PUNE	Community based model<br>PLHIV network led model
MANIPUR	BISHNUPUR	Community based model
	CHANDEL	Community based model
	CHURACHANDPUR	Community based model
	IMPHAL EAST	Community based model
MIZORAM	AIZAWL	Community based model
	CHAMPHAI	Community based model
	LUNGLEI	Community based model

State	District	Models implemented
	MAMIT	Community based model
NAGALAND	DIMAPUR	Community based model
	KOHIMA	Community based model
	MOKOKCHUNG	Community based model
	TUENSANG	Community based model
PUNJAB	AMRITSAR	Community based model
	JALANDHAR	Community based model
	LUDHIANA	Community based model
TAMIL NADU	CHENNAI	Community based model<br>Private practitioners' model
TELANGANA	HYDERABAD	Community based model<br>Private practitioners' model
	KHAMMAM	Community based model<br>Private practitioners' model
	WARANGAL	Community based model<br>Private practitioners' model
UTTAR PRADESH	AURAIYA	Community based model
	KANPUR	Community based model
	LUCKNOW	Community based model
	PRAYAGRAJ	Community based model
WEST BENGAL	KOLKATA	Community based model<br>Private practitioners' model
	NORTH 24PARAGANA	Community based model<br>Private practitioners' model

Supplementary Table S2: Questions, responses and dichotomisation of the primary outcomes

Outcome	Question	Possible responses	Dichotomisation for analysis
Acceptability for future use	The next time when you go for HIV testing, which method would you prefer?	1- HIV self-testing<br>2- Integrated Counselling and Test Centre (ICTC)<br>3- Community-based screening under TI<br>4- Private facility<br>5- Others (specify)<br>6- No response	1 - HIV self-testing<br>2- All other preferences<br>3 - No response
	Which HIVST kit will you prefer? (Only for participants who answered HIVST in the question above)	1 - Oral-fluid-based HIVST kit<br>2 - Blood-based HIVST kit<br>3 - No response	
Usability - Ease of using the kit	How easy did you find using the test?	1- Very easy<br>2- Easy<br>3- Neither easy nor difficult<br>4- Difficult<br>5- Very difficult<br>6- No response	1 - Very easy and easy -> Easy to use<br>2 - Neither easy nor difficult, difficult and very difficult -> Not easy to use<br>3 - No response -> Missing
Usability - Ease of interpreting the result	How easy was it to interpret the test result?	1- Very easy<br>2- Easy<br>3- Neither easy nor difficult<br>4- Difficult<br>5- Very difficult<br>6- No response	1 - Very easy and easy -> Easy to use<br>2 - Neither easy nor difficult, difficult and very difficult -> Not easy to use<br>3 - No response -> Missing

Outcome	Question	Possible responses	Dichotomisation for analysis
Willingness to pay	There is a possibility that in the future the HIVST kit you used as part of this study may not be provided for free to everybody. How much will you be willing to pay for an HIV self-testing kit? (Only for participants who preferred HIVST kits for future use)	1- Less than 100 rupees<br>2- Between 101 and 250 rupees<br>3- Between 251 and 500 rupees<br>4- More than 500 rupees<br>5- Not willing to pay<br>6- No response	1 - Not willing to pay -> Not willing to pay<br>2 - Willing to pay any amount -> Willing to pay<br>3 - No response -> Missing

Supplementary Figure S1: Directed acyclic graph (DAG) of ease of interpreting the HIV self-test result.

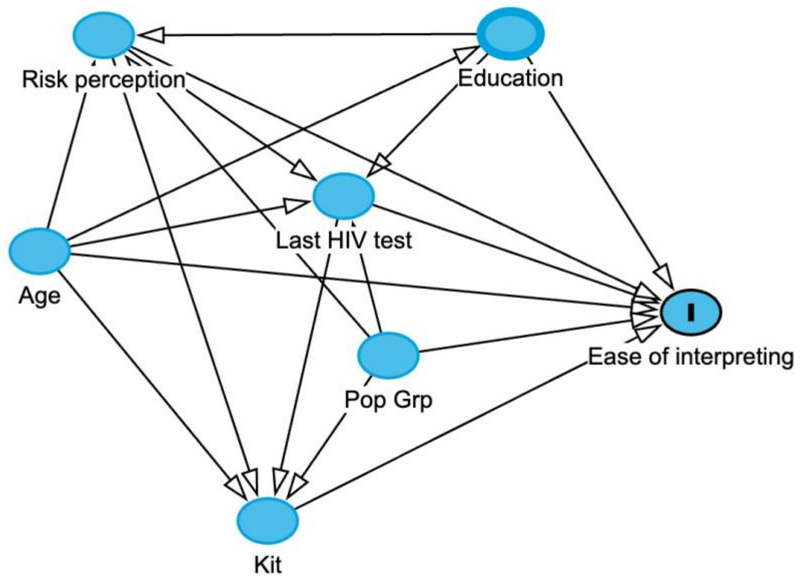

Supplementary Figure S2: Directed acyclic graph (DAG) of ease of use of HIV self-testing kit.

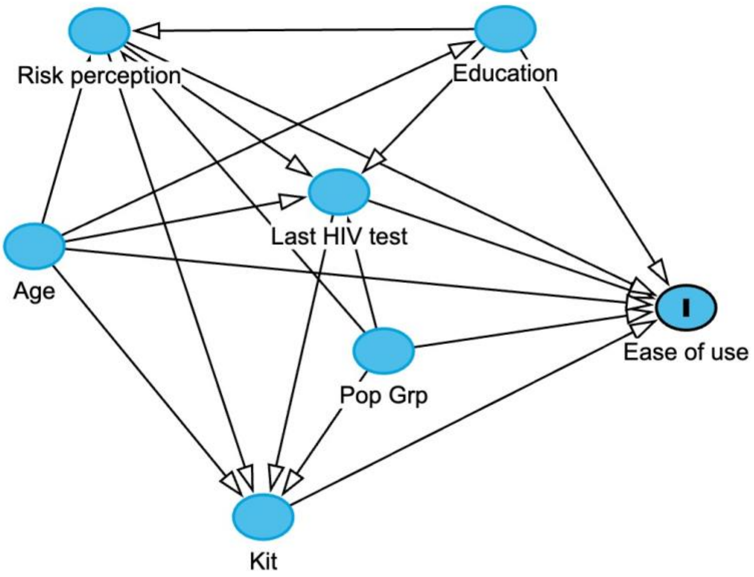

Supplementary Figure S3: Directed acyclic graph (DAG) of acceptance of HIV self-testing for next use.

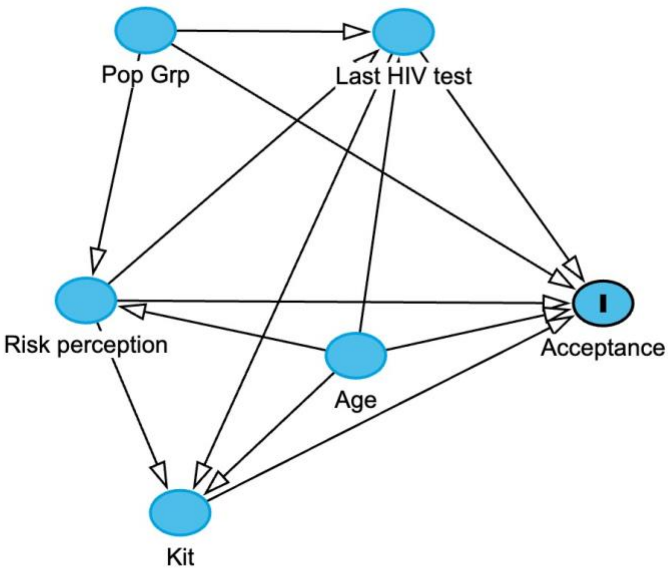

Supplement: Supplementary file 1 — Table S1: State and district‐wise HIVST distribution models implemented under the STAR III study in India Table S2: Questions, responses and dichotomization of the primary outcomes Figure S1: Directed acyclic graph (DAG) of ease of interpreting the HIV self‐test result. Figure S2: Directed acyclic graph (DAG) of ease of use of HIV self‐testing kit. Figure S3: Directed acyclic graph (DAG) of acceptance of HIV self‐testing for next use. [file JIA2-27-e26348-s001.pdf]
